# Gamma Radiation Imaging System via Variable and Time-Multiplexed Pinhole Arrays

**DOI:** 10.3390/s20113013

**Published:** 2020-05-26

**Authors:** Ariel Schwarz, Amir Shemer, Yossef Danan, Rachel Bar-Shalom, Hemy Avraham, Alex Zlotnik, Zeev Zalevsky

**Affiliations:** 1 Department of Electrical and Electronics Engineering, Azrieli College of Engineering, Jerusalem 9103501, Israel; arielsc@jce.ac.il (A.S.); yossefda@jce.ac.il (Y.D.); 2 Shaare Zedek Medical Center, Jerusalem 9103102, Israel; barshalom@szmc.org.il (R.B.-S.); ahemy@hadassah.org.il (H.A.); 3 Faculty of Engineering, Bar-Ilan University, Ramat-Gan 5290002, Israel; alex.zlotnik@gmail.com (A.Z.); Zeev.Zalevsky@biu.ac.il (Z.Z.)

**Keywords:** biomedical imaging, coded aperture imaging, image coding, image resolution, multipinhole collimators, nuclear medicine, pinhole collimators, SPECT

## Abstract

Biomedical planar imaging using gamma radiation is a very important screening tool for medical diagnostics. Since lens imaging is not available in gamma imaging, the current methods use lead collimator or pinhole techniques to perform imaging. However, due to ineffective utilization of the gamma radiation emitted from the patient’s body and the radioactive dose limit in patients, poor image signal to noise ratio (SNR) and long image capturing time are evident. Furthermore, the resolution is related to the pinhole diameter, thus there is a tradeoff between SNR and resolution. Our objectives are to reduce the radioactive dose given to the patient and to preserve or improve SNR, resolution and capturing time while incorporating three-dimensional capabilities in existing gamma imaging systems. The proposed imaging system is based on super-resolved time-multiplexing methods using both variable and moving pinhole arrays. Simulations were performed both in MATLAB and GEANT4, and gamma single photon emission computed tomography (SPECT) experiments were conducted to support theory and simulations. The proposed method is able to reduce the radioactive dose and image capturing time and to improve SNR and resolution. The results and method enhance the gamma imaging capabilities that exist in current systems, while providing three-dimensional data on the object.

## 1. Introduction

In the field of human clinical radionuclide imaging two major techniques are used. These techniques are known as single photon emission computed tomography (SPECT) and positron emission tomography (PET). Those techniques are invaluable especially for highly sensitive molecular imaging in the study of human diseases, the testing of new pharmaceuticals, the development of new imaging tracers and the understanding of biological mechanisms.

A gamma camera detector can determine the presence and position of an incident gamma photon, but obtains no information about the incidence direction. For this reason, in the SPECT technique system, a parallel hole collimator is positioned between the object and the gamma camera detector and so, only photons, which originate from specific directions, can continue on their path to the crystal detector. The parallel hole collimator is one of the principal methods available for making images with gamma radiation in nuclear medicine applications. The systems of gamma imaging do not include an imaging lens and thus, the imaging is performed using an array of channels positioned in close proximity to the inspected object, allowing projection of the object on the detector with no magnification. The parallel hole collimator SPECT is a 1:1 projection system and the projection image size is almost independent of the collimator distance. This is true both for 2D and 3D imaging using the SPECT technique 

The main disadvantages of this concept are as follows: i. the array of channels transmits only rays at zero degrees and thus the overall sensitivity efficiency is low (approx. 10^−6^); ii. since this imaging technique uses a projection, the field of view determines the size of the detector. Thus, for imaging a 50 cm planar image, one needs a detector of the same size. 

One approach to solve these problems is to use pinhole imaging with the gamma camera. A single pinhole collimator (typically with the shape of a double cone) is placed between the object and the gamma camera instead of the parallel holes collimator. For the pinhole collimator SPECT system, the sensitivity is inversely proportional to the square of the object-to-pinhole distance. However, the size of the projection on the gamma detector (which is relative to the object size) is determined by the ratio of the distances between the detector and the pinhole, and the pinhole and the object [1]. Consequently, using a pinhole collimator can collect magnified projections. This is an important property, since a projection acquired with a magnification is equivalent to a non-magnified projection acquired with a detector that has better resolution but smaller field of view. The size, shape and material of the pinhole are important to obtain the imaging characteristics.

To further increase the sensitivity of a pinhole SPECT system, using a multiple pinhole SPECT can allow one to obtain simultaneously captured projections from different angles. Moreover, those captures from different angles can also give an added value as well as the improvement in sensitivity, such as obtaining 3-D imaging from planar images. Each pinhole projection can be captured by a separate gamma camera, but it is also possible to capture the projections using one gamma camera by rotating the camera around the object, and in that way increasing the required scan time. There are many multipinhole SPECT designs, some rotate the detectors around the object, some rotate the object, and some have a stationary design with a multipinhole collimator. These stationary systems with simultaneous capture are suited for dynamic scans with short accumulation times. There are also many design parameters for multipinhole collimators, such as the number of pinholes, acceptance angle, pinhole diameter, the degree of focusing, magnification, and the amount of overlapping (multiplexing) of the pinhole projections on the detector. Further improvements in sensitivity can be obtained by focusing all pinholes to a small volume of interest as obtained in focused multipinhole SPECT systems. In order to reconstruct an image from overlapped projections in a multiplexed multipinhole SPECT systems, reconstruction algorithms (analytic or iterative) are used. For example, the iterative method of ordered subsets expectation maximization (OSEM) [2]. 

The first multipinhole collimator system used in nuclear medicine was the seven-pinhole collimator introduced by Vogel [3,4]. This collimator was designed to perform 3-D imaging from seven nonoverlapping pinhole projections rather than to improve sensitivity of the planar image. Ivanovic suggested a similar system with an optimized multipinhole collimator acquiring nonmultiplexed projections over a limited angular range [5]. Others have suggested stationary SPECT systems based on a larger number of single pinhole detectors [6,7,8,9]. The stationary imagers gather the projection data simultaneously within a short scan time, and are non-multiplexing pinhole systems (have no overlapping projections). Meikle and Wilson suggested systems that perform SPECT from multiplexed projection data, with overlapping pinhole projections consisting of one or more multipinhole detectors rotating around the object [10,11,12]. Wilson who used a synthetic collimator, introduced another method [13]. The synthetic collimation data was collected for only one direction but for different pinhole-to-detector distances. Many other multipinhole SPECT designs have been proposed, all differing over a range of properties [14,15,16,17,18,19,20,21,22,23,24,25,26,27,28,29]. 

Another solution to the above mentioned problems is related to the usage of coded aperture imaging (CAI). The use of a coded aperture was first introduced by Mertz and Young by using an off-axis Fresnel zone plate to image X-ray stars. The decoding was done by using coherent light [30]. Later on, Barrett et al. applied this method to nuclear medical imaging for planar and limited 3-D imaging of smaller objects. They used a lead grid in front of the object to reduce the background associated with CAI and in order to enable continuous imaging of the gamma-ray sources performed by the off axis zone plate [31,32]. A coded aperture consisting of a number of random pinholes, with an average transmission of 50% was suggested by Dicke for X-ray astronomy [33]. Many other research projects have tried to improve the X-ray and gamma ray imaging systems over the years [34,35,36,37,38,39,40,41,42,43]. The CAI technique has also been adapted by Accorsi to near field geometry of medical imaging [44,45,46]. Note that the common problem for all those suggested solutions based on multipinhole array and coded aperture is the loss of information due to narrow optical transfer function (OTF). The OTFs of a single pinhole and a multipinhole array have zero points that affect the final reconstruction.

## 2. The Imaging Concept

In order to solve the three problems mentioned above—resolution on one hand and sensitivity on the other, and without affecting the reconstruction due to the OTF zero points—we propose a novel approach of coded aperture with multipinhole arrays and time multiplexing for gamma imaging applications. This approach is based on our previous work that presented a lensless imaging concept with new approach of a multivariable coded aperture (VCA) design for far field and near field imaging [47,48]. In the present research, we have applied this approach to gamma radiation imaging applications. By using the time multiplexing approach, although each array in the time interval has zero points in the OTF, the overall array maintains higher spatial frequencies of the object. The usage of a time-variated multipinhole array, instead of a static multipinhole array with the same total number of holes, is essential. The time multiplexed array creates the complement encoding aperture with minimum loss of information and recovers the information loss of a static multipinhole array. In this configuration, we allow gamma planar imaging which is not just a 1:1 projection system. The main advantages of such a system are simplicity and flexibility that allow a wide range of design options. The pinhole array design determines the characteristics of the gamma imaging system, as well as the improvement factor of the sensitivity efficiency, and the signal to noise ratio (SNR) parameters. The proposed design shortens the accumulation time while preserving the same image quality. Additionally, it enables using the system for human clinical radionuclide imaging as well as for small-animal object imaging. The proposed concept is suitable for adding to existing SPECT gamma imaging systems. It also can be added to multipinhole SPECT systems to improve its performance.

**Figure 1:**
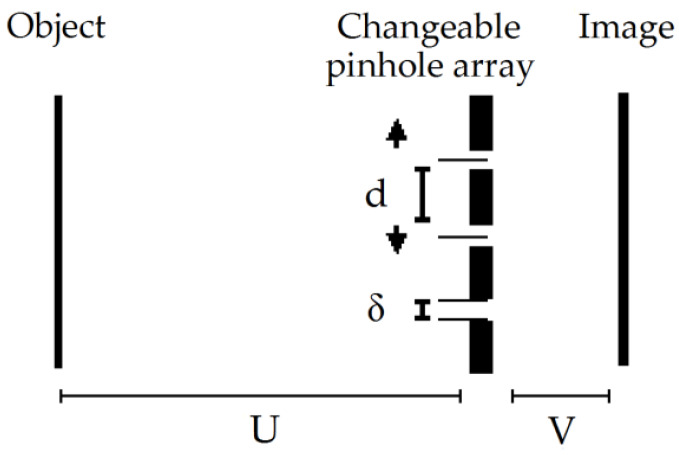
A schematic sketch of the proposed setup.

The final image is reconstructed after time multiplexing, i.e., it is obtained after properly processing a captured image during the multipinhole array variation (equivalent to a set of L captured images). The schematic sketch of the proposed system is given in Figure 1. The imaging system is composed of L pinhole arrays (one array for each time interval). Each array is designed differently and has different spacing d (the pitch of the holes in the array). Note that the distances between the pinholes in the same array are not necessarily equal. While changing the pinhole distances in each array, in every step of the coding part, the distance between the planes (U, V) and δ (the size of the holes) remains constant (see Figure 1). Hence, the pinhole array allows transmission of rays at all angles and not only rays propagating straight forward.

In order to understand the operational principle of the proposed configuration, let us first examine the case of a single pinhole. It is clear that in this case, an imaging is obtained (as in optics when a lens is in use) in contrast to the array of channels. The problem is that imaging using a single pinhole suffers from a tradeoff between low sensitivity efficiency in the case of a small pinhole or from low spatial resolution in the case of a large pinhole. 

The magnification ratio of such imaging system is:

(1) $$M=\frac{V}{U}$$

In the case of an array of pinholes being used, the same operational principle is obtained as before. However, this time the image obtained at the image plane consists of a plurality of image replications of the inspected object. In the case of an odd number of pinholes, there are equal distances between pinholes and a central pinhole, and the replication distances are proportional to the pitch d between the holes in the pinhole array. Thus, the resulted image obtained is

(2) $$s_{R}\left(x\right)=\overset{\frac{N-1}{2}}{\underset{n=-\frac{N-1}{2}}{\sum }}s\left(x+nd\right)$$

where *N* is the number of holes and *s*(*x*) is the image obtained with a single pinhole. The result is spatially low passed (i.e., image with reduced spatial resolution). This can be clearly seen by computing the spectrum of the spatial distribution of Equation (2):

(3) $$S_{R}\left(\mu \right)=\int^{\text{​}}s_{R}\left(x\right)e^{-2\pi ix\mu }dx=S\left(\mu \right)\cdot F\left(\mu \right)$$

where *S*(*µ*) and *F*(*µ*) are the following Fourier transforms:

(4) $$\begin{array}{l} S\left(\mu \right)=\int^{\text{​}}s\left(x\right)e^{-2\pi ix\mu }dx\\ F\left(\mu \right)=\overset{\frac{N-1}{2}}{\underset{n=-\frac{N-1}{2}}{\sum }}e^{-2\pi ind\mu } \end{array}$$

Therefore, *F*(*µ*) is basically a low pass filter that cuts off the high spatial frequencies in the imaged object. The cut off frequency is roughly proportional to

(5) $$\mu_{cutoff}=\frac{1}{Nd}$$

Thus, the smallest spatial feature being imaged is proportional to Nd in the image plane or Nd/M in the object plane.

Nevertheless increasing the number of holes in the array will increase the sensitivity efficiency, proportionally for 2-D array design. The point is that the resolution of *s*(*x*) is limited by a single pinhole. However, due to the summation, the overall resolution is reduced to the limit described by Equation (5). From the optics point of view (regardless of the detector resolution), we denote the wavelength by *λ*, the size of the pinhole by *δ* and the size of the smallest feature in the image plane by *ρ* and obtain the following resolution limitation equation:

(6) $$\rho =\frac{\lambda V}{\delta }$$

We intend to capture L images while changing the pinhole array between each capture and while trying to recover the original resolution of Equation (6) by applying matrix inversion. This will be our applied super resolved algorithm. 

By changing the pitch d of the holes in the pinhole array we get different filters, as we can see in the next set of equations:

(7) $$\begin{array}{l} S_{R}{}^{\left(1\right)}\left(\mu \right)=S\left(\mu \right)\cdot F^{\left(1\right)}\left(\mu \right)\\ S_{R}{}^{\left(2\right)}\left(\mu \right)=S\left(\mu \right)\cdot F^{\left(2\right)}\left(\mu \right)\\ \vdots \\ S_{R}{}^{\left(L\right)}\left(\mu \right)=S\left(\mu \right)\cdot F^{\left(L\right)}\left(\mu \right) \end{array}$$

and in general:

(8) $$S_{R}{}^{\left(l\right)}\left(\mu \right)=S\left(\mu \right)\cdot F^{\left(l\right)}\left(\mu \right)$$

where *S*_*R*_^(*l*)^(*µ*) is the Fourier transform of the set of images:

(9) $$S_{R}{}^{\left(l\right)}\left(\mu \right)=\int^{\text{​}}s_{R}{}^{\left(l\right)}\left(x\right)e^{-2\pi ix\mu }dx$$

The filter causes a loss of information and this will damage the reconstruction. In the following example one can see the areas in the filter (frequency domain) where there will be loss of information. For N = 3 and equal d we get:

(10) $$s_{R}\left(x\right)=s\left(x-d\right)+s\left(x\right)+s\left(x+d\right)$$

(11) $$\begin{array}{l} S_{R}\left(\mu \right)=\int^{\text{​}}s\left(x\right)e^{-2\pi ix\mu }dx=\\ S\left(\mu \right)\left[e^{-i2\pi \mu d}+1+e^{i2\pi \mu d}\right]=S\left(\mu \right)\cdot F\left(\mu \right) \end{array}$$

(12) $$F\left(\mu \right)=\left[e^{-i2\pi \mu d}+1+e^{i2\pi \mu d}\right]$$

where *s_R_* and *S_R_* are the obtained spatial and spectral distributions, respectively. 

**Figure 2:**
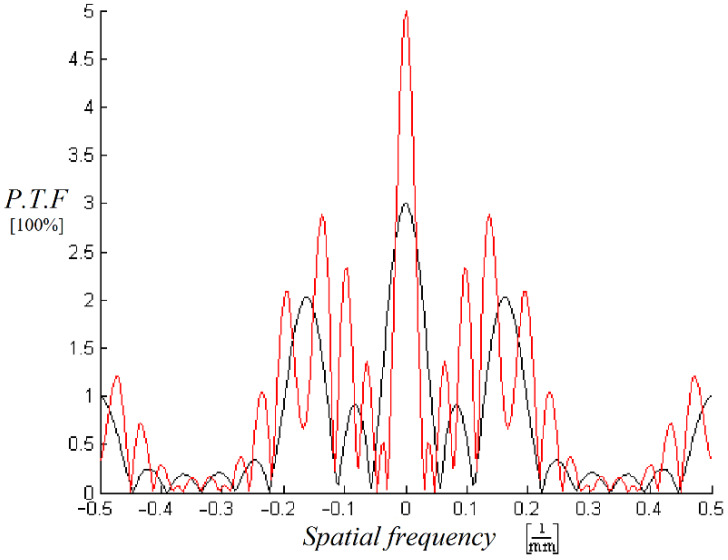
The *F*(*µ*) filter: Fast Fourier Transform of the Low Pass Filter and the spectral areas with loss of information (zero points in the spectral area). The pinhole array filter with N = 3 (black) and with N = 5 (red).

Here we can see areas in the frequency domain where some of the object information is lost and cannot be reconstructed (Figure 2). In the figure below, P.T.F. stands for pinhole transfer function and it represents a relative unit that is normalized to the transfer function of a single pinhole equal to one (100%) for a single pinhole. 

In order to preserve all the spatial frequencies of the original object image, we change three free parameters in our configuration; one is the spatial position of the pinholes in each array; the second is the number of pinholes in each set of arrays; and the third is the capture intervals for each pinhole array set. We choose the parameters in such a way that there will be no frequency loss in the filters’ sum *G*(*µ*):

(13) $$\overset{L}{\underset{l=1}{\sum }}S_{R}{}^{\left(l\right)}\left(\mu \right)=S\left(\mu \right)\cdot \overset{L}{\underset{l=1}{\sum }}F^{\left(l\right)}\left(\mu \right)$$

(14) $$G\left(\mu \right)=\overset{L}{\underset{l=1}{\sum }}F^{\left(l\right)}\left(\mu \right)$$

We denote by *S_R_*^(*l*)^(*μ*) the l image out of the L images, in the sequence obtained at the detector. 

Note that in our configuration, we decided to focus on performing 1-D reconstruction although it can be broadened to 2-D configuration. Furthermore, the time interval is equal, although this is not obligatory and one can change the time intervals, and by doing so control the energy level contributed by each array in the set to the overall sum *G*(*µ*).

Now, by using the inverse of the filters sum *G*(*µ*), one can extract the original image *s*(*x*) from the set of images *s_R_*^(*l*)^(*x*). It will result in better sensitivity efficiency and SNR. The algorithm used, is performed in the spatial Fourier plane as follows:

(15) $$S\left(\mu \right)=\left[\overset{L}{\underset{l=1}{\sum }}S_{R}{}^{\left(l\right)}\left(\mu \right)\right]\cdot G{\left(\mu \right)}^{-1}$$

Hence, the sensitivity efficiency improves and it is equal to:

(16) $$\eta =\frac{N}{L}\cdot \frac{\pi R_{array}^{2}}{\pi R_{one}^{2}}=\frac{N}{L}\cdot \frac{\delta_{array}^{2}}{\delta_{one}^{2}}$$

The first term is the ratio between number of total holes in the arrays (*N*) and number of arrays in the set (*L*). Note, that L is related to time axis. The second term is the ratio between a single pinhole area in the array system and the pinhole area in one pinhole system. Reducing the value of *δ_array_* in the second term improves resolution while affecting the radiation activity. 

Note, that in array systems aiming to improve sensitivity efficiency, regardless of resolution, the pinhole size can be equal to one pinhole system. Thus, the second term equals 1. Moreover, note that the resolution improvement of the pinhole array system, when compared to 1:1 system, is proportional to the magnification M. 

However, the inverse filter has some disadvantages in the sense of noise amplification, and thus other filters and algorithms can be used in the system according to the proper application, for example, matched filter, Wiener filter, Tikhonov regularization, Richardson–Lucy algorithm and more [49,50,51,52].

## 3. Results

### 3.1. Simulation Results

#### 3.1.1. The Multipinhole Array Designs MATLAB Simulation 

In order to investigate the parameters of the variable multipinhole array systems, the 1-D array designs were simulated. 

**Figure 3:**
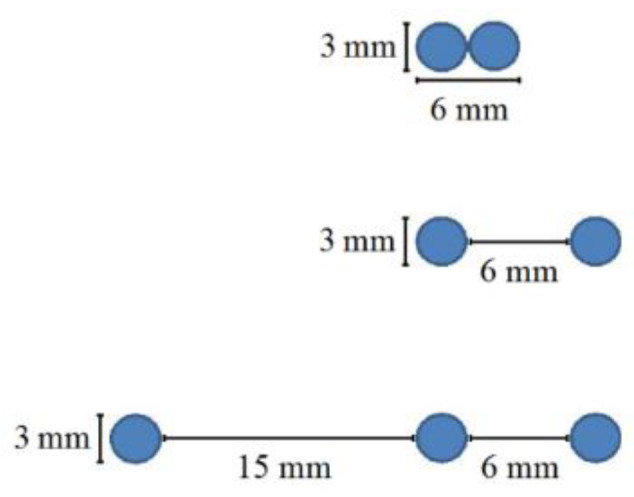
The three array states of 1-D pinhole design that were simulated and tested in the experiment.

Over time, in the multiplex pinhole array configuration, the number of pinholes in an array was changed during the accumulation process. The configuration consisted of two coupled pinholes in the first array, two pinholes in the second and three pinholes in the third array (Figure 3).

**Figure 4:**
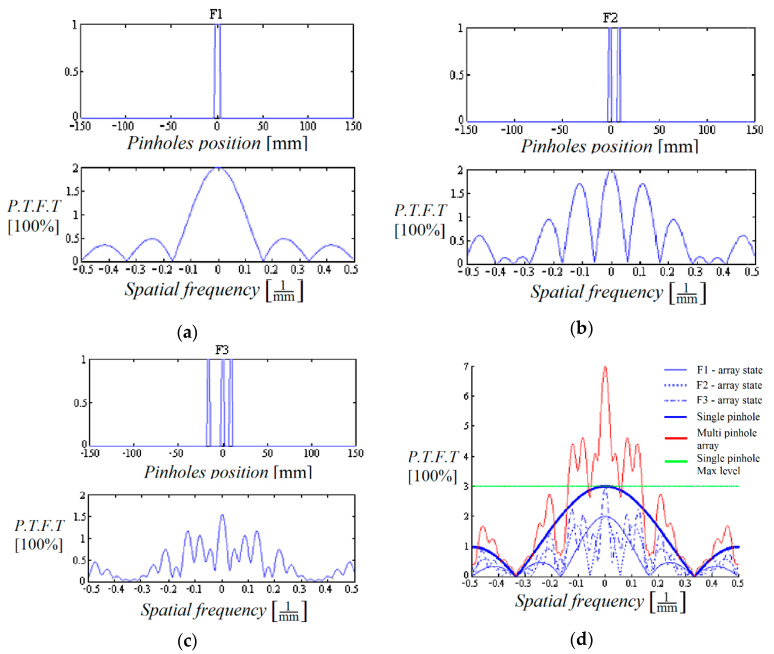
Simulation of the designed setup: The three 1-D pinhole arrays (space and frequency domains); (**a**) F1 is the filter related to the first pinhole array; (**b**) F2 is the filter related to the second pinhole array; (**c**) F3 is the filter related to the last pinhole array; (**d**) The sum of the three filters (top red) as compared to a single pinhole (top blue). The straight green line is the maximum level of the one pinhole reference level.

The pinhole array 1-D designs were designed by simulations performed according to the conditions that were mentioned above. Our considerations for choosing the positions and number of pinholes were first to prevent zero points in the spectral filters’ sum *G*(*µ*). The second consideration was to make sure that the filters’ sum *G*(*µ*) transmission value was higher than the reference filter in the region of interest (Figure 4). Note that in 1-D design, although there are two narrow spectral bands where the sum *G*(*µ*) is lower in respect to the one pinhole system, the spectral information is preserved, but with higher noise. However, the overall outcome is still better than the results of the one pinhole system. In the figures below P.T.F.T. stands for P.T.F. × 1 time unit—that is, the pinholes’ transfer function computed in relative units. One P.T.F.T. unit is the transmission equal to that of a single pinhole, multiplied by the accumulation time of one image from the set of L images. 1 time unit = total accumulation time/L.

#### 3.1.2. Geant4 Simulation

The designed setup system was tested in simulation, and its system parameters were as mentioned above. The simulations were performed using Geant4 10.3 toolkit for Monte-Carlo, high-energy particle transport [53,54]. This software package is composed of tools, which accurately simulate the passage of particles through matter.

The simulation is, in the main, composed of three parts: the particle sources, the pinholes and the detector. The particle sources simulation uses the G4GeneralParticleSource, which is part of the Geant4 toolkit. Specifically, it allows the specifications of the spectral, spatial and angular distribution of the primary source particles. A set of gamma particle sphere sources with energy of 40 Kev were simulated. The angular distribution was confined to the pinholes’ mask direction to save computational time. The pinholes’ shape was completed by defining a box made of tungsten with tube shapes made of vacuum in the pinholes’ location. The detection was performed by Geant4 sensitive detector, which counts every particle entrance to the detector’s pixels during the event action. A 50 × 50 pixel detector with a pixel dimension of two square mm was simulated. In order to shorten the running time, we divided the simulation of the gamma sources to several separate simulations and ran them in parallel using GNU Parallel shell tool for executing jobs in parallel and summing the results [55].

**Figure 5:**
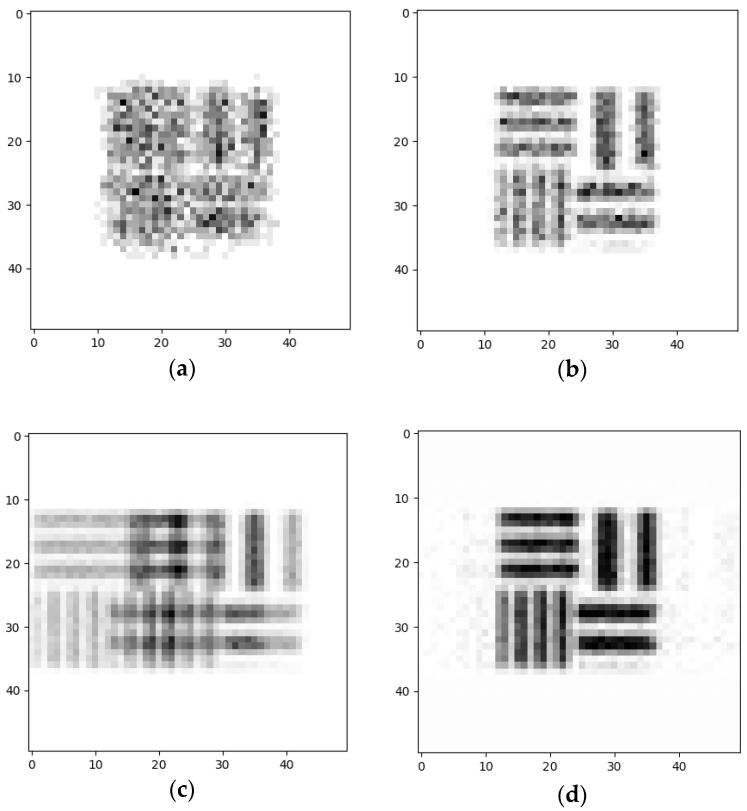
Simulation results for different systems (same scan time): (**a**) one pinhole system image, 4.45 mm diameter; (**b**) one pinhole system image, 2 mm diameter; (**c**) the captured image of the 1-D pinhole array system (no reconstruction); (**d**) the reconstructed image of the 1-D pinhole array system.

In the decoding part, a Wiener filter was used for the reconstruction algorithm. The simulation and the calculation obtained from the results present the ability to reconstruct an object from several images and to improve the sensitivity efficiency and SNR, as can be seen in Figure 5.

The sensitivity efficiency of the multipinhole system was found in both simulations and in comparison with the one pinhole system in order to improve the SNR. The theoretical improvement in the SNR between the two systems is shown in Table 1. 

### 3.2. Experimental Results

The SPECT system in the experiments was the GE Discovery NM/CT 670 and Infinia. The nuclear detector characteristics of the system are as follows: 3/8″ (9.5 mm) crystal thickness: 59 circular PMT’s—53 × 3″ (76 mm) and 6 × 1.5″ (38 mm); UFOV (usable field of view): 54 × 40 cm ± 0.5 cm; intrinsic spatial resolution: 3.6 mm (3.9 mm for Infinia). The material of all arrays was lead with tungsten pinhole inserts. Hole insert diameter (δ): 4.45 mm; magnification: 1; object to array distance (U): 14 cm; array to detector distance (V): 14 cm; acceptance angle: 75°.

**Figure 6:**
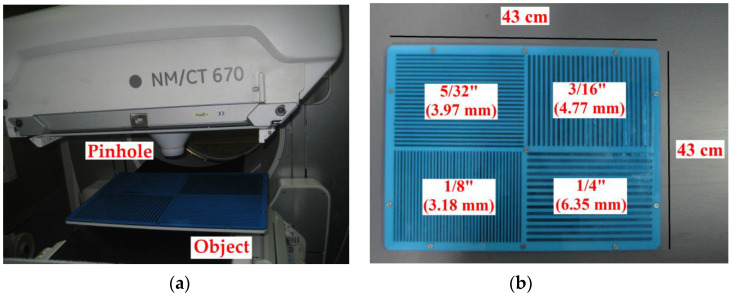
(**a**) The object (bar phantom lead plate) in the gamma radiation nuclear imaging SPECT system; (**b**) the bar phantom overall size: 16-7/8″ × 16-7/8″ (43 × 43 cm), bar widths: 1/4″, 1/8″, 3/16″ and 5/32″ (6.35, 3.18, 4.77, 3.97 mm).

**Figure 7:**
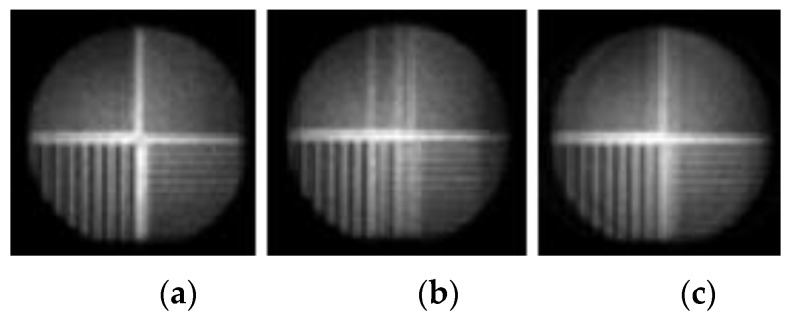
Gamma radiation nuclear imaging system’s results: (**a**) the reference image from a one pinhole system; (**b**) the captured image of the 1-D pinhole array system; (**c**) The reconstructed image of the 1-D pinhole array system with better sensitivity efficiency (improvement factor of 2.333) and better SNR.

The next stage involved experimental validation performed with a gamma radiation system that was available in the General Electric Healthcare laboratories in Haifa, Israel and in the Nuclear Medicine unit at the Shaare Zedek Medical Center, Jerusalem Israel. The system was configured and controlled according to simulation parameters using super position of the one pinhole. The object was a special bar phantom made of lead (Figure 6). The real system results with the bar phantom object are shown in Figure 7. The results show that in the case of the 1-D pinhole array system (1,2,3 configuration), compared with the one pinhole reference system, better sensitivity efficiency is obtained, with an improvement factor of 2.333 and better SNR with an improvement factor of $\sqrt{2.333}$. The SNR ratio is calculated based on comparison between the increases in signal in the variable pinhole method to the signal in the one pinhole system while using the same conditions. The results were achieved without reducing resolution and at the same scan time. In order to emphasize the improvement of the proposed multipinhole system, the following test was performed: first, an object (coin) was imaged in the one pinhole system with a short accumulation time (18 s), so that the object was not recognized in the captured image. Then, the same image capture was performed in the multipinhole array system with same accumulation time (18 s). Here, however, the object was well recognized and the result looks the same as an image obtained in a one pinhole system but with a long accumulation time (42 s).

**Figure 8:**
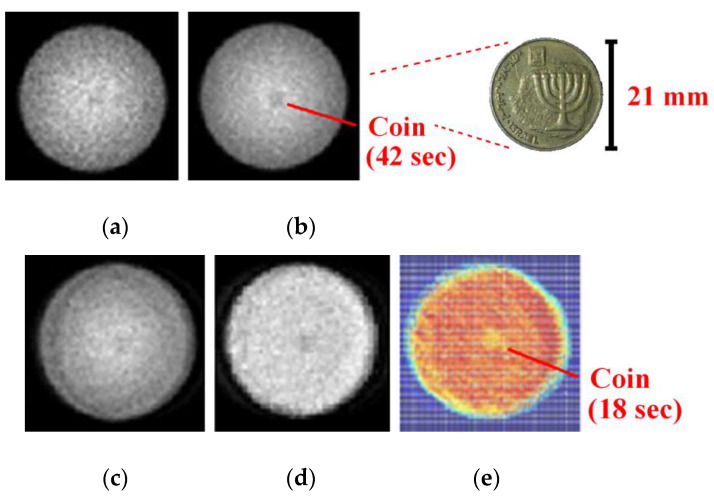
Coin test results—comparing scan time of the two systems: (**a**) one pinhole system with a coin not recognized after short accumulation time (18 s scan); (**b**) one pinhole system image obtained with long accumulation time (42 s scan); (**c**) the recognized coin in the variable multipinhole array system after short accumulation time (18 s scan); (**d**,**e**) reconstructed image in the variable multipinhole array system after dome effect correction.

Thus, the time scan improvement factor (of 2.333) was obtained using the proposed concept. Uniformity correction was also performed and the dome effect of the gamma detector was corrected. The experimental results are shown in Figure 8. The relative CNR (contrast to noise ratio) and the SNR comparison between the systems are shown in Table 2 and Table 3. 

The improvements achieved enable the shortening of the accumulation time by the factor correlated to the multipinhole system parameters. Note that the resolution is related to the pinhole diameter, thus we compare the performance based on using a smaller pinhole—2 mm (higher resolution but lower sensitivity due to the low counting rate) and a large pinhole—4.45 mm (lower resolution but higher sensitivity due to the high counting rate). It was compared with the multipinhole system using a 2 mm pinhole (higher resolution and higher sensitivity due to the high counting rate). In order to show resolution improvement with a multipinhole array system, while preserving the same gamma radiation activity and scan time, the following test with a two-system comparison was performed:

(17) $$\frac{\pi R_{1}^{2}}{\pi R_{2}^{2}}=\frac{4.45^{2}}{2^{2}}=4.95$$

**Figure 9:**
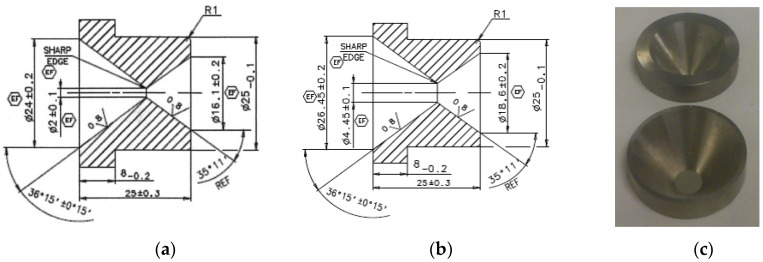
(**a**) The 2 mm pinhole insert design; (**b**) The 4.45 mm pinhole insert design; (**c**) The 2 mm pinhole insert (top) and the 4.45 mm pinhole insert (bottom), both made of tungsten.

**Figure 10:**
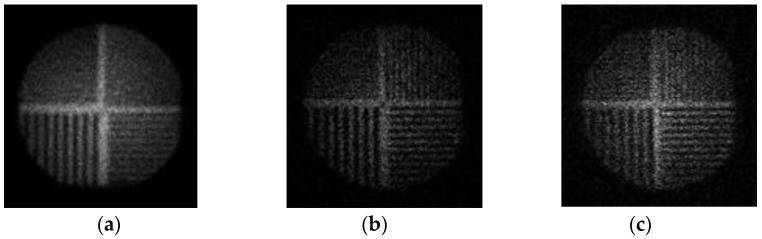
Sensitivity efficiency and SNR improvement-same scan time: (**a**) single pinhole—4.45 mm diameter, low resolution; (**b**) Single pinhole—2 mm diameter, low sensitivity efficiency; (**c**) multipinhole array—2 mm diameter, high resolution and sensitivity efficiency.

The object was a bar phantom lead plate. The one pinhole system used had a hole insert diameter (δ) of 4.45 mm while the multipinhole array system had a hole insert diameter (δ) of 2 mm (Figure 9). The improvement factor of the 1-D array design compares to a 2 mm single pinhole system is 2.333 (total number of seven pinholes/three arrays). The gamma radiation activity ratio between the 2 mm system and the 4.45 mm system is according to the pinholes’ area ratio: The resolution improvement results are presented in Figure 10. The same pinhole array configuration but in 2-D design for each array will give an improvement factor of 5.667.

## 4. Conclusions

In this paper, we have presented a method for improving sensitivity efficiency and SNR in the gamma imaging system used for nuclear medicine applications. This was completed using known designs of multiple pinhole array coded aperture. We demonstrated that the desirable improvement can be achieved without changing the image resolution and without increasing the image acquisition time. The proposed concept was validated numerically and experimentally.

## Figures and Tables

**Table 1:** Calculated simulation results—signal to noise ratio (SNR): SNR improvement comparison showing noise counts for equal signal counts in the two systems. Note that the noise in the single pinhole system is larger by factor of $\sqrt{2.333}$ in comparison to noise in the 1,2,3 pinhole array system.

	One Pinhole System [Avg. Counts] Accumulation Time: 180 s			Multipinhole System [Avg. Counts] Accumulation Time: 180 s		
Object to Background Ratio 1:1.2	Signal	Noise (Std)	SNR	Signal	Noise (Std)	SNR
Object	13.56837806	3.88248836	3.49476336	31.57960458	5.62920882	5.60995436
Background	11.16794390	3.34176248	3.34193228	26.12460000	5.16068550	5.06223447

**Table 2:** Calculated experiment results—SNR: systems’ SNR comparison of coin images with short accumulation time.

	One Pinhole System [Avg. Counts] Accumulation Time: 42 s		Multipinhole System [Avg. Counts] Accumulation Time: 18 s	
	Signal	Noise (Std)	Signal	Noise (Std)
Object	4.061305	0.313631	4.242393	0.222412
Background	4.475837	0.2993	4.564631	0.26209

**Table 3:** Calculated experiment results—relative contrast to noise ratio (CNR): Systems’ relative CNR of the coin experiment. Improvement in CNR is achieved enabling to shorten the time by ~2.3.

	One Pinhole System		Multipinhole Array System
**Accumulation time (s)**	18	42	18
**Relative CNR**	1	1.43	1.38
